# Using Virtual Reality to Induce and Assess Objective Correlates of Nicotine Craving: Paradigm Development Study

**DOI:** 10.2196/32243

**Published:** 2022-02-15

**Authors:** Weichen Liu, Gianna Andrade, Jurgen Schulze, Neal Doran, Kelly E Courtney

**Affiliations:** 1 Department of Computer Science and Engineering University of California, San Diego La Jolla, CA United States; 2 Department of Psychiatry University of California, San Diego La Jolla, CA United States; 3 Veterans Affairs San Diego Healthcare System La Jolla, CA United States

**Keywords:** nicotine, craving, cue-exposure, virtual reality, attentional bias, pupillometry, development, smoking, addiction, eye-tracking

## Abstract

**Background:**

Craving is a clinically important phenotype for the development and maintenance of nicotine addiction. Virtual reality (VR) paradigms are successful in eliciting cue-induced subjective craving and may even elicit stronger craving than traditional picture-cue methods. However, few studies have leveraged the advances of this technology to improve the assessment of craving.

**Objective:**

This report details the development of a novel, translatable VR paradigm designed to both elicit nicotine craving and assess multiple eye-related characteristics as potential objective correlates of craving.

**Methods:**

A VR paradigm was developed, which includes three Active scenes with nicotine and tobacco product (NTP) cues present, and three Neutral scenes devoid of NTP cues. A pilot sample (N=31) of NTP users underwent the paradigm and completed subjective measures of nicotine craving, sense of presence in the VR paradigm, and VR-related sickness. Eye-gaze fixation time (“attentional bias”) and pupil diameter toward Active versus Neutral cues, as well as spontaneous blink rate during the Active and Neutral scenes, were recorded.

**Results:**

The NTP Cue VR paradigm was found to elicit a moderate sense of presence (mean Igroup Presence Questionnaire score 60.05, SD 9.66) and low VR-related sickness (mean Virtual Reality Sickness Questionnaire score 16.25, SD 13.94). Scene-specific effects on attentional bias and pupil diameter were observed, with two of the three Active scenes eliciting greater NTP versus control cue attentional bias and pupil diameter (Cohen *d*=0.30-0.92). The spontaneous blink rate metrics did not differ across Active and Neutral scenes.

**Conclusions:**

This report outlines the development of the NTP Cue VR paradigm. Our results support the potential of this paradigm as an effective laboratory-based cue-exposure task and provide early evidence of the utility of attentional bias and pupillometry, as measured during VR, as useful markers for nicotine addiction.

## Introduction

Craving for substances is considered essential for understanding the pathogenesis and maintenance of addiction, as highlighted by the incentive salience model [1,2] and for the inclusion of craving as a criterion for substance use disorder in the Diagnostic and Statistical Manual of Mental Disorders (5th edition; DSM-5) [3] and the International Classification of Diseases (10th edition; ICD-10) [4]. Nicotine craving specifically has been shown to predict lapse to cigarette smoking following cessation [5,6] and is frequently identified by individuals as an important barrier to quitting and maintaining abstinence [7]. Thus, craving represents a clinically important phenotype of nicotine addiction [8] with great potential for intervention.

Accurate assessment of craving is essential for the identification, management, and treatment of nicotine and tobacco product (NTP) use and the use of other substances [9,10]. In human laboratory studies, craving for nicotine and other abused substances is commonly measured using the cue-exposure paradigm. The translational value of the cue-exposure paradigm to the naturalistic environment is predicated on the observation that relapse to drug use is often precipitated by exposure to drug-related cues that provoke craving [11,12]. However, naturalistic cues can be very complex and involve a number of contextual factors that are difficult to replicate in laboratory-based cue-exposure paradigms [13], limiting their ability to invoke a *true* craving state [9,14]. New technologies such as virtual reality (VR) afford the opportunity to increase the ecological validity of cue-exposure paradigms through the implementation of interactive and immersive presentations of cues within the typical context of use (eg, the presence of others within a setting where the substance is commonly taken), greatly enhancing our ability to invoke craving in the laboratory [9]. Studies using VR cue-exposure have found great support for its effectiveness in inducing subjective, and in some cases objective, craving for tobacco [15-17], as well as alcohol [13,18], cannabis [19], and methamphetamine [20].

Furthermore, despite decades of research, the field of addiction has yet to establish reliable, objective measures of craving. A number of objective correlates of craving have been investigated, including psychophysiological (eg, heart rate variability and skin conductance) and neurological (eg, functional magnetic resonance imaging and blood oxygenation level dependent activation) measures with varying success [14,21]. Attentional bias, or the ability of drug cues to capture the attention of the user, can be conceptualized as a behavioral marker of incentive salience [22] and represents an objectively measurable and clinically important phenomenon for the study of addiction. Attentional bias toward smoking cues has been previously demonstrated among regular tobacco smokers [23-26], and importantly, it has been related to the risk of subsequent relapse following smoking cessation [27].

Multiple theoretical models suggest that cue-induced subjective craving and attentional bias reflect closely linked underlying processes [1,28,29]. Not surprisingly, measures of attentional bias have been shown to correlate with subjective craving [30]. However, the method of assessment appears to be key—direct measures of attention such as the assessment of eye movement, exhibit larger craving correlations [30] and greater reliability [31-34] than indirect measures such as reaction time. Assessment within naturalistic settings has also independently improved the reliability [35] and validity [36] of attentional bias measurement; yet, the naturalistic constraints of these methods prohibit advanced clinical application of these paradigms. New technological advances in VR implementation allow for the assessment of eye movement in a noninvasive and cost-effective manner and demonstrate early success in distinguishing smokers and nonsmokers on the basis of eye fixations to smoking cues in a virtual world [26].

Spontaneous eye blink rate (EBR) represents another, much less studied, potential objective correlate of cue-induced craving. EBR has been closely linked with striatal dopaminergic function and has been advanced as a reliable [37], more cost-effective, and minimally invasive alternative to positron emission tomography (PET) to assess dopaminergic functioning [38]. Dopamine release in the basal ganglia (including the striatum) inhibits the spinal trigeminal complex, leading to increased EBRs, as demonstrated in both rat and human trials [39]. In line with this theory, preclinical research has shown that direct dopaminergic agonists and antagonists increase [40] and decrease EBRs [39-41], respectively. Furthermore, a PET study in monkeys found a strong positive correlation between EBRs and dopamine (D_2_) or D_2_-like (D_3_) receptor availability in the striatum [42]. Given the observed modulation of striatal dopamine during cue-elicited substance craving [43,44], it may be possible to detect NTP cue-induced dopamine changes through EBR measurement. Nonetheless, no studies to date have investigated this hypothesis.

Lastly, pupillometry represents an additional potential objective craving correlate. Pupil dilation is an indirect measure of norepinephrine (NE) release from the locus coeruleus and is associated with reward processing [45], including sensitivity to rewards [46], and engagement of cognitive resources [47]. Pupillary responses also seem to index changes in the allocation of attention and have been advanced as an ideal measure for related constructs that may not pass the threshold for overt behavior or conscious appraisal [48]. To our knowledge, only one study has investigated pupillometry as a measure of response to substance cue-exposure. Kvamme et al [49] found that pupillary bias toward alcohol versus neutral cues, but not subjective craving reports, predicted relapse to alcohol use in a sample of detoxified patients with alcohol dependence [49], suggesting that cue-induced changes in pupillometry may ultimately serve as a useful biomarker for addiction research and clinical care.

This study was intended to outline the methods underlying the development of a novel VR-NTP cue-exposure paradigm with embedded eye-characteristic assessments. Preliminary analyses on a pilot sample of participants are also provided as a proof of concept for the potential utility of this paradigm for the induction of subjective craving in the laboratory, assessment of potential biomarkers of craving (ie, attentional bias, EBR, and pupillary dilation), and prediction of NTP use behaviors.

## Methods

### NTP Cue VR Paradigm Development

The NTP Cue VR paradigm uses a virtual reality environment built using Unity. The HTC Vive Pro Eye VR headset (HTC) was used to enable VR capabilities and collect eye-related data. HTC’s SRanipal SDK [50] was used in conjunction with Tobii’s (Tobii Technology) Tobii XR SDK [51] to provide access to various data from the eye tracker. Specifically, Tobii XR SDK handled object selections, determining what participants were looking at, with its Gaze-to-Object Mapping (G2OM) algorithm, while the rest of the data were retrieved from the SRanipal SDK. The participants were free to move around (via teleportation) and interact with various objects within the VR environment using 2 hand-held Vive controllers. Surveys (a visual analogue scale [VAS] with a range of 0-100) assessing depressed mood and anxiety were presented at the start of the paradigm (following the initial training and test scenes) and additional surveys assessing subjective craving (“How much are you craving nicotine right now?”) and scene relevance (“How relevant was that scene to your own life?”) were presented between scenes within the headset. A VAS survey was chosen as the in-task measurement of subjective craving owing to its high face-validity, ability to capture the dynamic fluctuations in craving [52], and low burden on participants, especially over frequently repeated assessment. Survey responses were made by adjusting a slide bar using one of the controllers. Participants were instructed to “Just explore everything around you until the scene changes” and “During the task, we will be measuring what you pay attention to, and we will be asking you to rate your craving level between each scene.”

**Figure 1:**
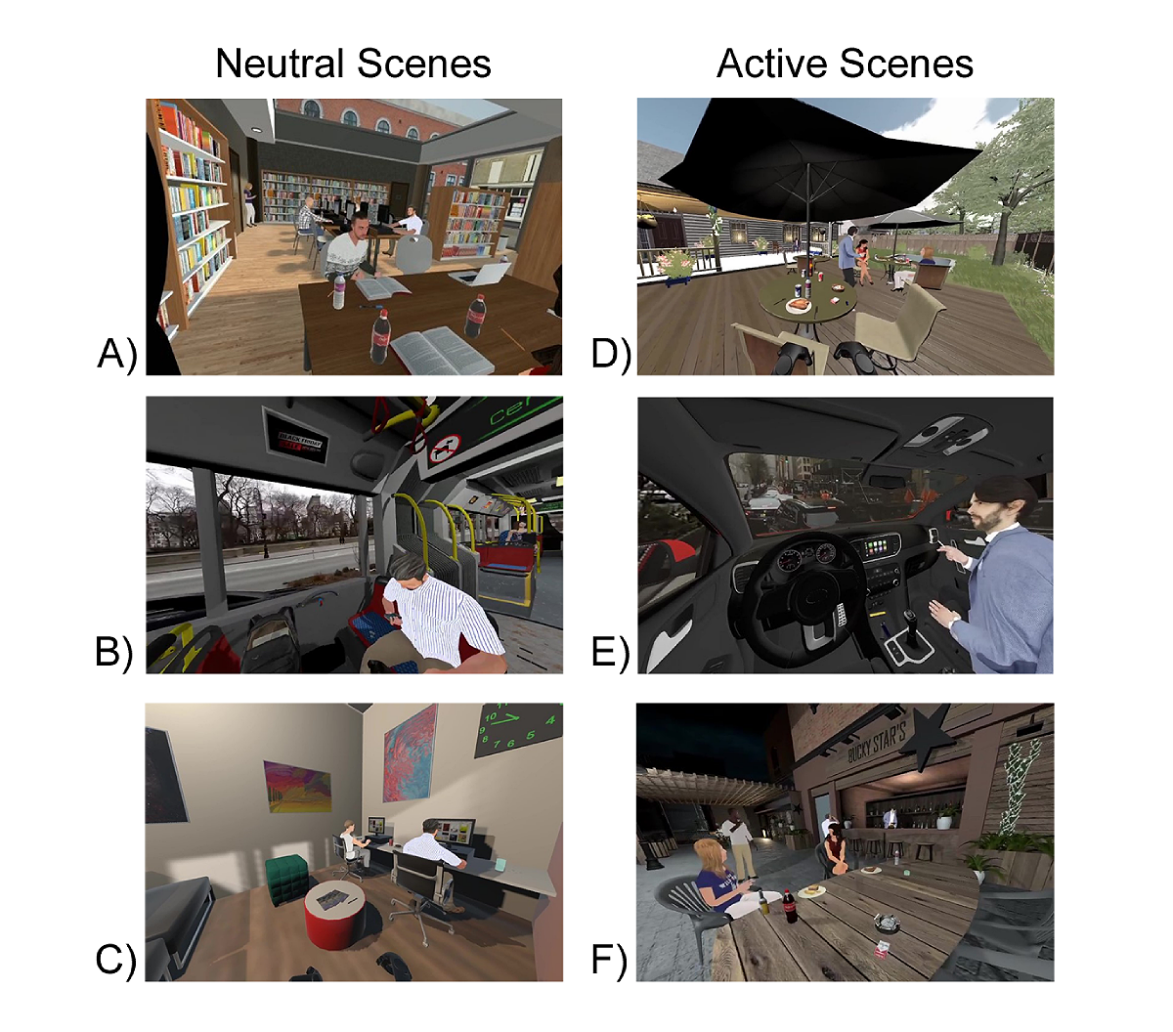
Screenshots of the 6 scenes from the NTP Cue VR paradigm. Neutral scenes include the (A) Library, (B) Bus, and (C) Waiting Room. Active scenes include the (D) Outdoor BBQ, (E) Driving, and (F) Patio. NTP: nicotine and tobacco product; VR: virtual reality.

Three Active scenes (Driving, Patio, and Outdoor BBQ) and three Neutral scenes (Bus, Waiting Room, and Library) were developed and included in the final paradigm (see Figure 1 for screenshots of the scenes). The Active scenes include NTP-related cues, while in the Neutral scenes, all cues are neutral. Active cues include ashtrays, lighters, JUUL devices, cigarettes (individual and packs), Puffbars, hookahs, as well as the presence of human models engaged in smoking or vaping behaviors. Neutral cues (eg, water bottles, cellphones, pens or pencils, magazines, and candies) vary depending on the scene context. All cues are interactable such that the participants are able to pick up, throw, and collide the items with other items in the scene. All scenes (Active and Neutral) include the presence of at least one animated human model. Smoke and vapor effects are incorporated with the animated human models in the Active scenes to increase the immersiveness of the experience. All scenes include background music and audio effects consistent with the scene and the participants’ interaction.

### NTP Cue VR Paradigm Procedure

The NTP Cue VR paradigm begins with 3 “test scenes,” which are approximately 3 minutes in duration, depending on participant comfort and abilities with the VR hardware. The first scene is the Practice Room. This is a square room with cubes systematically placed around corners of the room. The participants are asked to gaze at each of the boxes to confirm that the eye-tracking is functioning as intended. Then, the participants are asked to practice using the controllers to teleport to 4 different locations in the room. The second scene is the Practice Slider room, which instructs the participants how to answer the survey questions and provides the opportunity to practice adjusting the slider to answer the scales. The third test scene is the Blink Calibration room. In this scene, the participants are asked to blink 5 times after being prompted by an audio signal. The purpose of this room is to collect pupil diameter data when the participants actively blink to assist with increasing the accuracy of blink detection algorithms. Following the completion of the initial test scenes, the 2 mood surveys are presented, and the 6 scenes (3 Active and 3 Neutral) are pseudorandomized within scene type such that the general scene order is maintained (Active, Neutral, Active, Neutral, Active, and Neutral). The participants are then placed in each scene for 5 minutes. The entire paradigm is approximately 30 minutes in duration.

### Data Collection

There are 2 types of data recorded within each scene, regular time series and event-based data that is recorded at event onset. Regular time series data are collected at every 10-millisecond interval (100 Hz), independent of the frame time. The following data are recorded periodically: (1) timestamp, (2) raw gaze intersection point, (3) position and forward direction of the participants' headset, and (4) pupil diameter and eye openness (calculated by SRanipal SDK). The following events and corresponding timestamps are recorded when they occur: (1) blinks, including number of blinks and the object of gaze at the time of the blink; (2) button presses on the controller, including time, button pressed, and object of interaction (if applicable); and (3) object of gaze when eye gaze switches to a new object.

### Gaze Statistics Calculation

Raycasting from the eye position was initially used to enable object selection in the direction of gaze. However, this raycasting method did not perform well in our experiments, especially for very small objects, owing to the limited precision and accuracy of the eye tracker, microsaccades, etc. Therefore, for small objects of interest, we utilized the G2OM algorithm provided by the Tobii XR SDK, which is a machine learning–based object selection algorithm that aims to improve small object– and fast-moving object–tracking. Based on our testing, this algorithm improved object selection over the naïve method but still lacked selection quality. Thus, to further improve object selection, we introduced an additional mechanism to “lock” the object selection when an object is manipulated such that whenever a participant actively picks up a virtual object, the object selection algorithm will always select the picked object until the participant releases the object. If the participant is not interacting with an object, the G2OM algorithm is employed, or if no small objects are within the field, naïve raycasting is employed.

To calculate eye-gaze statistics toward active and neutral cue objects, 4 dictionaries corresponding to 4 different types of objects (Active, Neutral, Miscellaneous, and Background) are initialized prior to the start of participant involvement in the paradigm. These dictionaries are then used to store the cumulating gaze fixation or dwell time durations as values for individual objects belonging to each object and type. When a participant gazes at an object, the object is searched in the dictionary on the basis of its name and type. If the object was encountered before, the current fixation time is added to its cumulative fixation time. If the object had not been encountered before, a new entry is created for the object. The fixation time is then calculated as the difference between the timestamp of current entry and that of the next line of entry.

Following the completion of the paradigm, total fixation time indices are produced, which reflect the sum of values within each dictionary (Active, Neutral, Miscellaneous, and Background). The mean fixation time indices are also created, which reflect the total fixation time divided by the number of objects (number of keys) gazed at by the participant.

### Blink Detection

Initially, we tested a measurement of eye openness, as calculated by the HTC SRanipal SDK, as an indicator for blink detection. However, given the lack of established thresholds of eye openness for blink detection, we instead chose to rely on estimates of pupil diameter. Consistent with previous studies, an eyeblink is herein defined as complete eyelid closure with the pupil covered for 50-500 milliseconds [53,54]. For any given timepoint, we consider a missing pupil diameter reading as a possible complete eyelid closure where the pupil is completely covered by the eyelid. These eye closure durations are blink candidates. If either pupil is covered for less than 50 milliseconds, the candidate is discarded as it is more likely owing to noise or an eye tracker limitation. If either pupil is covered for more than 500 milliseconds, the candidate is also discarded as this is more consistent with a microsleep [54,55]. Using this blink detection definition, the blink count for the majority of the current participants fell within 12-40 blinks per minute, which appears to align with the consensus of spontaneous blink rates in the literature [55-58].

### Participant Recruitment and Screening Procedures

Participants for this ongoing study are recruited through flyers and web-based (eg, Facebook, Craigslist, and San Diego Reader) advertisements posted in the San Diego community. Interested individuals call the laboratory and complete a telephone-screening interview to determine initial eligibility. Inclusion criteria for the ongoing study are the following: (1) age >18 years, (2) nondaily (average use on 4-27 days per month in the past 3 months) or daily NTP use (average use on 7 days per week in the past 3 months), and (3) an NTP use history of ≥1 year. Exclusionary criteria are the following: (1) medical or psychiatric history affecting brain development (ie, history or treatment of neurologic disorders, severe head trauma with loss of consciousness for >2 minutes, or current severe DSM-5 psychiatric disorders other than tobacco use disorders), (2) nonfluency in English, (3) visual problems that may make task completion difficult (eg, severe motion sickness, blindness, and glasses).

Eligible participants are then invited for the in-person laboratory assessment and instructed to bring their NTP products with them for use immediately after the assessment to control for effects related to expectations of imminent substance availability [59]. They are asked to abstain from cannabis and alcohol use for at least 24 hours, and from NTP use for at least 1 hour, prior to testing.

### Ethical Considerations

Upon arrival to the laboratory, participants receive a full explanation of the study procedures and provide written, informed consent. The study protocol was approved by the University of California, San Diego Human Protections Program institutional review board (protocol 180719) and is in accordance with the Helsinki Declaration of 1975, as revised in 2000.

### Psychological Measures

Following consent procedures, participants undergo an extensive clinical interview and complete several self-report questionnaires covering demographic, psychological health (Mini International Neuropsychiatric Interview [MINI] for DSM-5 [60]), and substance use (90-day Timeline Follow-Back [TLFB] [61], PATH Tobacco Dependence [TD] [62], Customary Drinking and Drug Use Record [CDDR] [63], and Tobacco Craving Questionnaire-Short-Form [TCQ-SF] [64]) domains. The TLFB has high test-retest reliability for intervals ranging from 30 to 360 days prior to the interview date, with an intraclass correlation coefficient=0.92 for “Total number of cigarettes smoked per interval” [65]. Thus, past 90-day NTP use episode count from the TLFB was used in the quantitative analyses presented below. All study interview and self-report data were collected and managed using REDCap electronic data capture tools hosted at the University of California, San Diego.

Participants then undergo the NTP Cue VR paradigm, which includes repeated (postscene) assessments of subjective nicotine craving and scene relevance to the individual participant (VAS; see *NTP Cue VR Paradigm Development*). Upon completion of the paradigm, additional assessments on VR-related outcomes such as VR presence (Igroup Presence Questionnaire [IPQ] [66]) and VR-related simulator or motion sickness (Simulator Sickness Questionnaire [SSQ] [67]) are administered. The IPQ total score was calculated using a simple averaging method to obtain a single average perceived presence score ranging 0-100. Similarly, the SSQ was scored in concordance with procedures outlined to assess VR-specific sickness (Virtual Reality Sickness Questionnaire [VRSQ] [68]), which involves a simple averaging method to obtain a single average sickness score with a range of 0-100.

### Statistical Analysis of Pilot Data

These analyses include the first 31 participants to complete the study protocol; however, data were missing for some subjects on a subset of indices owing to technological difficulties (as indicated by the degrees of freedom for each test presented in the results section). Owing to safety restrictions related to COVID-19, no biological verification of abstinence was conducted. Group differences are not being investigated in the present pilot analyses since the goal of this study is to describe the development and general validity of the paradigm and to maximize statistical power. Statistical analyses were conducted using a repeated measures (ie, paired samples) *t* test (2-tailed) or Pearson correlation framework. The threshold of significance was set at *P*<.05 for all analyses. SPSS Statistics for Windows (version 27; IBM Corp) software was used for all analyses.

## Results

### Results Overview

Demographic information is presented in Table 1. In general, the sample is predominantly male (61%) and White (61%), and 61% had no or very limited (one time) previous experience with VR.

**Table 1:** Sample demographics (N=31).

Variable			Value
Age (years), mean (SD)			30.77 (16.33)
Sex (male), %			61.3
Ethnicity (White), %			61.3
Education (college level), %			74.2
**Previous experience with virtual reality, n**			
	Never	12	
	Once	7	
	A few times	9	
	Many times	3	
Nicotine and tobacco product use days (in the past 90 days), mean (SD)			60.10 (33.39)
Nicotine and tobacco product use episodes (in the past 90 days), mean (SD)			772.29 (1008.20)
Tobacco Craving Questionnaire score at baseline, mean (SD)			105.32 (9.46)
**VR^a^ presence (Igroup Presence Questionnaire score), mean (SD)**			
	Spatial presence	65.74 (16.46)	
	Involvement	62.08 (21.59)	
	Experienced realism	52.22 (23.87)	
	Total	60.05 (9.66)	
**VR-related sickness (Virtual Reality Sickness Questionnaire score), mean (SD)**			
	Oculomotor	16.94 (14.60)	
	Disorientation	15.56 (15.43)	
	Total	16.25 (13.94)	

^a^VR: virtual reality.

### Subjective Craving

**Figure 2:**
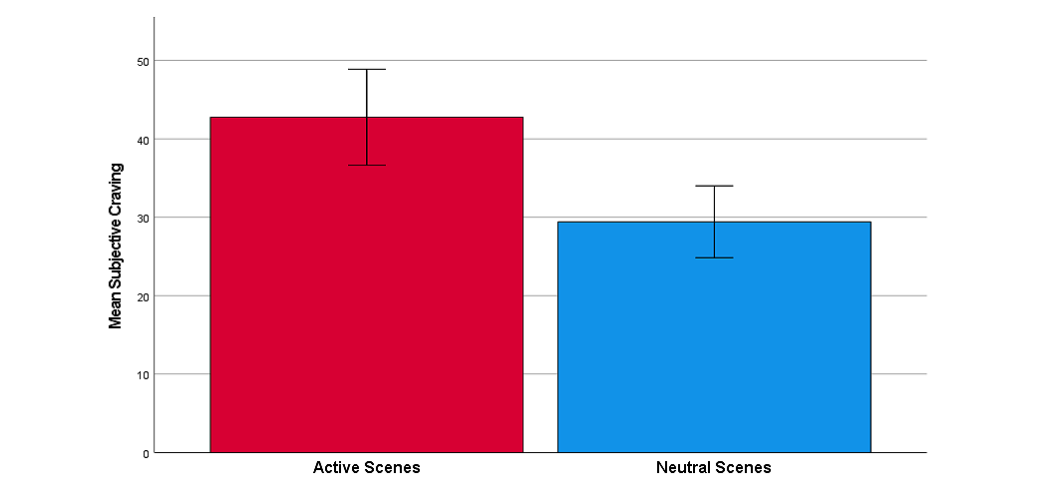
Mean subjective craving rating averaged across Active and Neutral scenes. Error bars indicate an SE of 1.

The paired samples *t* test, which investigated subjective craving during the paradigm, revealed a significant effect of scene condition on craving (*t*_30_=4.24, *P*<.001; Cohen *d*=0.76, 95% CI 0.36-1.16), with Active scenes (mean 42.77, SD 34.07) eliciting greater subjective craving than Neutral scenes (mean 29.42, SD 25.54; Figure 2). Pairwise comparisons tested among all Active scenes revealed that craving ratings were greater after the Driving scene (mean 48.77, SD 35.67) than after the Outdoor BBQ scene (mean 43.50, SD 35.79; *P*=.01) and the Patio scene (mean 40.00, SD 33.69; *P*=.01); yet, no difference in ratings was observed between the Outdoor BBQ and Patio scenes (*P*=.33).

### Attentional Bias

**Figure 3:**
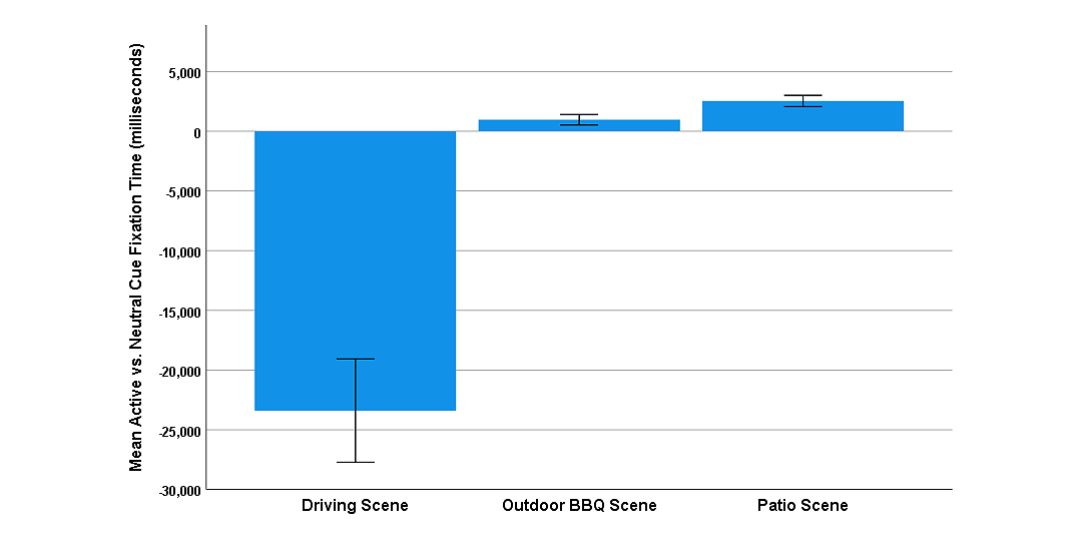
Mean Active versus Neutral cue fixation time (in milliseconds) within the 3 Active scenes. Error bars indicate an SE of 1.

The paired samples *t* test, which investigated eye-gaze fixation time during the paradigm, revealed a significant effect of cue-type on fixation time during the Active scenes (*t*_30_=–4.76, *P*<.001; Cohen *d*=–0.85, 95% CI –1.26 to –0.44), with greater mean fixation time toward Neutral cues (mean 12,888.52, SD 6314.20 milliseconds) compared to Active cues (mean 5807.98, SD 3002.78 milliseconds). Additional *t* tests within each Active scene (see Figure 3) revealed a greater Active (mean 4364.32, SD 2541.85 milliseconds) versus Neutral (mean 1962.32, SD 812.64 milliseconds) cue fixation time in the Patio scene (*t*_29_=5.05, *P*<.001; Cohen *d*=0.92, 95% CI 0.49-1.35), and a greater Active (mean 3060.69, SD 2183.26 milliseconds) versus Neutral (mean 2111.13, SD 972.85 milliseconds) cue fixation time in the Outdoor BBQ scene (*t*_29_=2.24, *P*=.03; Cohen *d*=0.41, 95% CI 0.03-0.78). However, we observed a lower Active (mean 10,238.44, SD 6037.01 milliseconds) versus Neutral (mean 34,723.50, SD 19,114.72 milliseconds) cue fixation time in the Driving scene (*t*_30_=–5.83, *P*<.001; Cohen *d*=–1.05, 95% CI –1.48 to –0.60).

### Pupil Diameter

**Figure 4:**
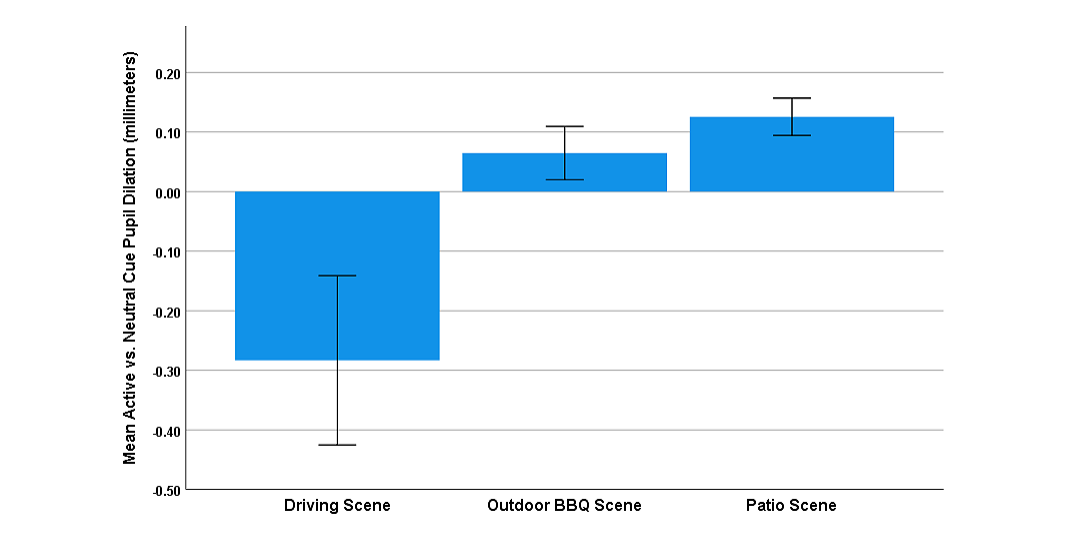
Mean Active versus Neutral cue pupil diameter (mm) within the 3 Active scenes. Error bars indicate an SE of 1.

The paired samples *t* test, which compared mean pupil diameters, revealed a smaller pupil diameter in response to Active cues (mean 3.87, SD 0.78 mm) than for Neutral cues (mean 3.97, SD 0.71 mm; *t*_28_=–2.01, *P*=.05; Cohen *d*=–0.37, 95% CI –0.75 to 0.01) averaged across Active scenes. Additional *t* tests within each Active scene (see Figure 4) revealed a greater Active (mean 3.95, SD 0.63 mm) versus Neutral (mean 3.83, SD 812.64 mm) cue pupil diameter in the Patio scene (*t*_27_=3.95, *P*<.001; Cohen *d*=0.75, 95% CI 0.32-1.16), and a trend for greater Active (mean 3.78, SD 0.61 mm) versus Neutral (mean 3.71, SD 0.59 mm) cue pupil diameter in the Outdoor BBQ scene (*t*_27_=1.60, *P*=.12; Cohen *d*=0.30, 95% CI –0.08 to 0.68). As observed for attentional bias, a lower Active (mean 4.16, SD 1.13 mm) versus Neutral (mean 4.43, SD 0.80 mm) cue pupil diameter was observed in the Driving scene (*t*_28_=–2.07, *P*=.05; Cohen *d*=–0.38, 95% CI –0.76 to –0.003).

### Spontaneous Eye-Blink (EBR)

The paired samples *t* test revealed no significant differences in EBR during Active and Neutral scenes (*t*_30_=0.49, *P*=0.62; Cohen *d*=0.09, 95% CI –0.26 to 0.44). Within Active scenes only, pairwise comparisons revealed that the Outdoor BBQ scene (mean 197.86, SD 96.80) was associated with a greater EBR than the Patio scene (mean 173.83, SD 76.90; *P*=.04; Cohen *d*=0.41, 95% CI 0.02-0.78). No differences were observed between the Driving scene (mean 193.47, SD 85.98) and the Outdoor BBQ (*P*=.53; Cohen *d*=0.12, 95% CI –0.24 to 0.47) or Patio scene (*P*=.21; Cohen *d*=0.24, 95% CI –0.13 to 0.60).

### Relationship to NTP Subjective Craving and Use

**Figure 5:**
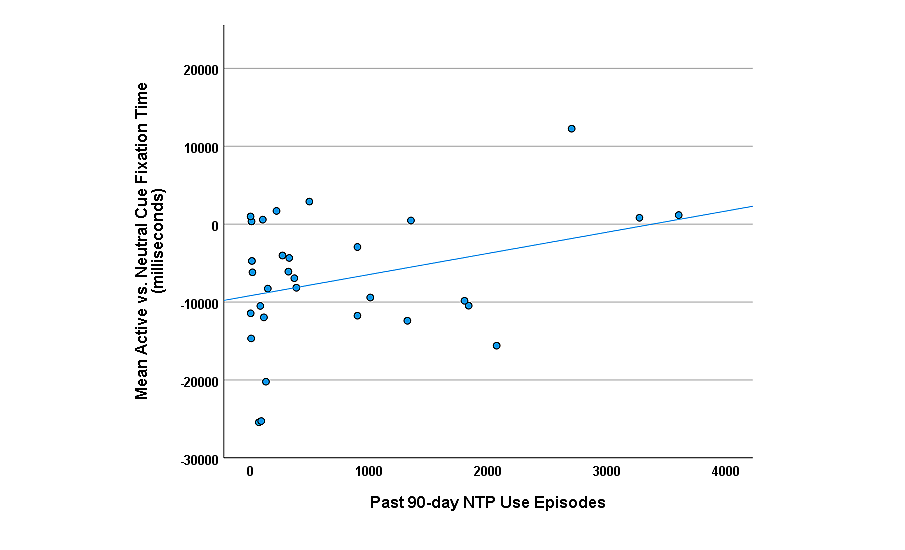
Scatter plot depicting the linear relationship between mean Active versus Neutral cue fixation time (in milliseconds) averaged across the 3 Active scenes and past 90-day nicotine and tobacco product (NTP) use episodes.

Exploratory Pearson correlations were investigated to provide an initial estimate of the potential for these objective metrics to serve as an indicator of subjective craving and past NTP use. Attentional bias (mean Active vs Neutral Cue fixation time across Active scenes), pupil diameter, and EBR were not found to significantly correlate with in-task subjective craving ratings (attentional bias: *r*_Driving_=–0.09, *r*_Patio_=0.16, *r*_Outdoor BBQ_=0.26, *P*>.05 for all; pupil diameter: *r*_Driving_=0.16, *r*_Patio_=0.01, *r*_Outdoor BBQ_=0.06, *P*>.05 for all; EBR: *r*_Driving_=0.12, *r*_Patio_=0.28, *r*_Outdoor BBQ_=0.19, *P*>.05); however, attentional bias was found to positively correlate with past 90-day NTP use episodes at a trend level (*r*=0.33, *P*=.06; see Figure 5). Similar positive correlations were observed for each scene separately (*r*=0.19-0.31). No relationships were observed between past 90-day NTP use and pupil diameter (*r*=–0.27, *P*=.17) or EBR (*r*=–0.05, *P*=.79).

## Discussion

### Principal Findings

This report describes our approach to the development of a novel NTP cue VR paradigm designed to simultaneously induce and assess potential eye-based objective correlates of nicotine craving in naturalistic and translatable virtual settings. The preliminary statistical analyses support the potential of this paradigm in its ability to induce subjective craving while instilling a moderate sense of presence in the virtual world and only low levels of VR-related sickness.

The preliminary results outline a potential context-specific effect of NTP-related attentional bias and pupil dilation in this pilot sample. Consistent with the literature on attentional bias [23-26] and pupil dilation [49], we observed greater Active NTP versus Neutral control cue-related effects in 2 of the 3 Active scenes (Patio and Outdoor BBQ). The similarity observed in the pattern of effects between attentional bias and pupil dilation provides early evidence of a potential cross-validation of these metrics. No effects were observed for the EBR metric; however, the size of this effect, if present at all, may be smaller than we are currently able to detect with the limited sample.

The observed reversal of attentional bias and pupil dilation toward neutral cues in the Driving scene warrants further investigation, given the large effect size. Potential explanations for this include the presence of especially engaging neutral cues in the Driving scene, as a 360° video of a busy city street is presented in the background, which participants report as entertaining to watch. Despite the overall bias toward neutral cues reflected in the global attentional bias metric, and within the Driving scene alone, participants with greater attentional bias toward NTP cues (even if negative) were found to endorse greater NTP use in the previous 90 days. This effect appears to be driven by the higher-frequency NTP users in our sample and is consistent with the literature supporting the validity of attentional bias as a clinically important indicator of nicotine addiction [27]. Additional analyses are planned to assess direct and indirect relationships between scene eye-related outcomes and relevance to the individual, scene-specific craving level, randomization of scenes, engagement with specific cues, and NTP use groups (ie, nondaily vs daily NTP users) once more data are collected.

### Strengths and Limitations

This pilot study has several strengths and limitations. Strengths include the development of a cutting-edge VR cue-reactivity task that incorporates the latest technological advances in graphic design to increase translatability to the real-world and simultaneous assessment of multiple potential eye-related indices of cue-reactivity in a 3D virtual environment. Limitations include the absence of biological verification to confirm self-reported NTP use and the inability to investigate NTP use profiles in the analyses owing to limited power. Importantly, given the limited sample size, we caution against over interpretation of our results. It remains unknown whether the absence of significant results, particularly with respect to the correlations between objective eye-related indices and subjective craving ratings, are the result of limited power to detect these relationships or true independence of these indices. However, we believe that the general pattern of scene-related effects on attentional bias and pupil dilation are encouraging and warrant further study. The identification of reliable objective correlates (“biomarkers”) of craving would allow for greater examination of the underlying neurobiological processes involved, and inform new avenues for the development of psychological and pharmacological treatments.

### Conclusions

To our knowledge, this is the first attempt to investigate eye-tracking indices (attentional bias, pupillometry, or EBR) within a VR substance cue-exposure paradigm. Taken together, the results of this preliminary data analysis suggest that this paradigm may prove useful for laboratory-based studies of NTP cue-reactivity and provide a platform for further investigation of eye-based markers of psychophysiological processes that may subserve the subjective craving experience. Once thoroughly tested and validated, this paradigm could function as a translatable platform for which experimental manipulations and craving interventions could be tested.

## Abbreviations

CDDR: Customary Drinking and Drug Use Record
D_2_: dopamine
DSM-5: Diagnostic and Statistical Manual of Mental Disorders (5th edition)
EBR: spontaneous eye blink rate
G2OM: Gaze-to-Object Mapping
ICD-10: International Classification of Diseases (10th edition)
IPQ: Igroup Presence Questionnaire
MINI: Mini International Neuropsychiatric Interview
NE: norepinephrine
NTP: nicotine and tobacco product
PET: positron emission tomography
SSQ: Simulator Sickness Questionnaire
TCQ-SF: Tobacco Craving Questionnaire-Short-Form
TD: PATH Tobacco Dependence
TLFB: 90-day Timeline Follow-Back
VAS: visual analog scale
VR: virtual reality
VRSQ: Virtual Reality Sickness Questionnaire
